# Supplementary material for: Leptospira transcriptome sequencing using long-read technology reveals unannotated transcripts and potential polyadenylation of RNA molecules
Source: Microbiol Spectr. 2023 Oct 20;11(6):e02234-23. doi: 10.1128/spectrum.02234-23 (PMC10715090; doi:10.1128/spectrum.02234-23)
Supplement: Supplemental figures — Fig. S1, S2, S3, S4. [file spectrum.02234-23-s0001.pdf]

***Leptospira* transcriptome sequencing using long-read technology reveals unannotated transcripts and potential polyadenylation of RNA molecules**

## Supplementary Figures

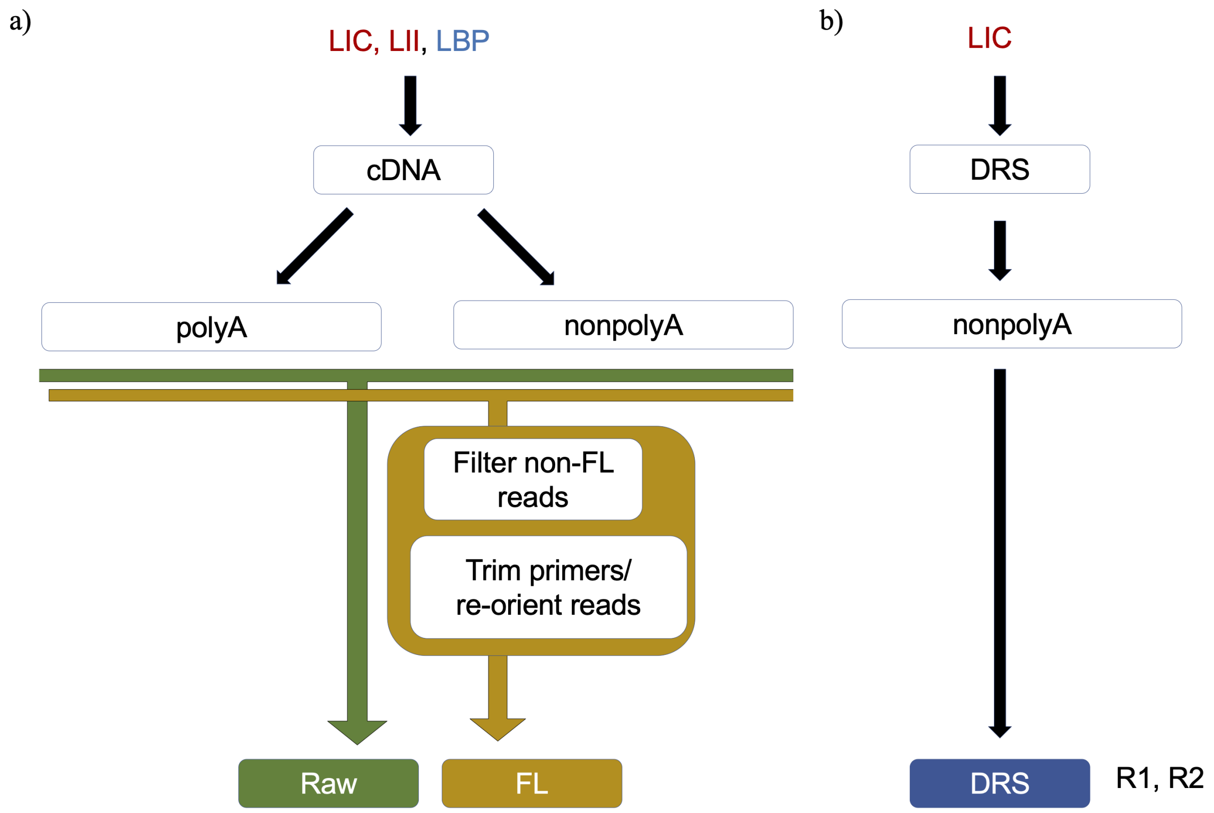


**Figure S1. Schematic process of sequencing process.** a) Three *Leptospira* strains from two different species (*L. interrogans* & *L. biflexa*) were sequenced using ONT's direct cDNA sequencing (cDNA) protocol with enzymatic polyadenylation (polyA) and without (nonpolyA) during library preparation (red: pathogenic strains (LIC: *L. interrogans* Copenhageni and LII: *L. interrogans* Icterohaemorrhagiae); blue: non-pathogenic strain (LBP: *L. biflexa* Patoc)). Two different cDNA datasets were generated using different processing protocols. The first dataset is composed by raw reads that were generated directly from basecalling and demultiplexing (raw, green). The second dataset was generated with the identification of the FL reads, primers trimmed, and reads oriented from the raw dataset (FL, orange). b) Two replicates (R1, R2) of LIC samples were also sequenced with ONT's DRS sequencing protocol (DRS) without polyadenylation.

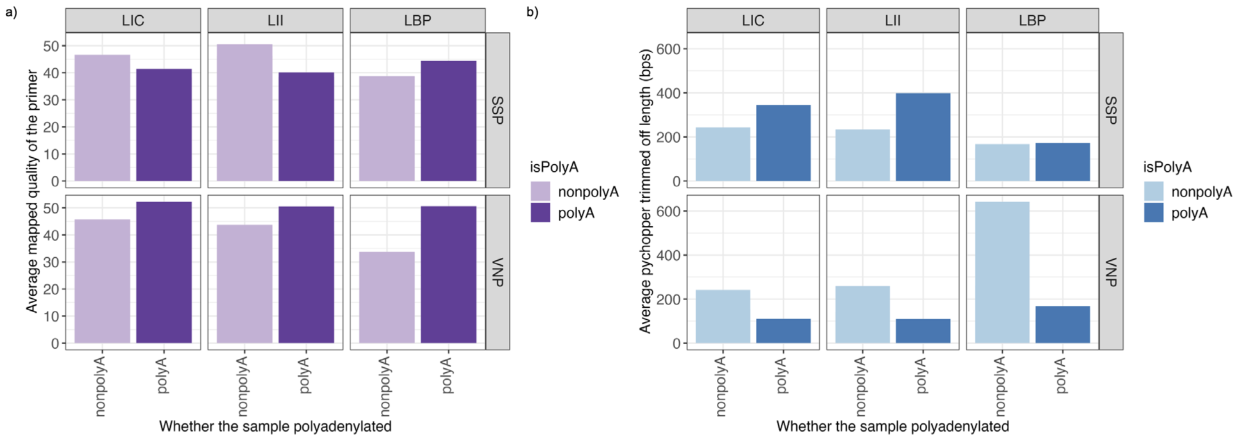


**Figure S2. Average mapping quality of the forward (SSP) and the reverse (VNP) primers.** These primers were identified from the raw sequenced cDNA reads and the average trimmed off length from the ends of the identified primers. a) The average mapping qualities of the identified SSP (top) and VNP (bottom) primers from all the identified full length (FL) reads reported by Pychopper (the FL read identification software) were calculated for each sample. The light purple bars show the average mapping qualities of the primers identified from the reads of the non-polyadenylated cDNA samples and the dark purple bars show those for the reads of the polyadenylated samples. b) The average trimmed off sequence length in number of base pairs (bp) by the Pychopper from the identified SSP (top) and VNP (bottom) primers. The light blue bars show the average trimmed off length from the non-polyadenylated cDNA reads and the dark blue bars show those trimmed off from the reads of the polyadenylated samples.

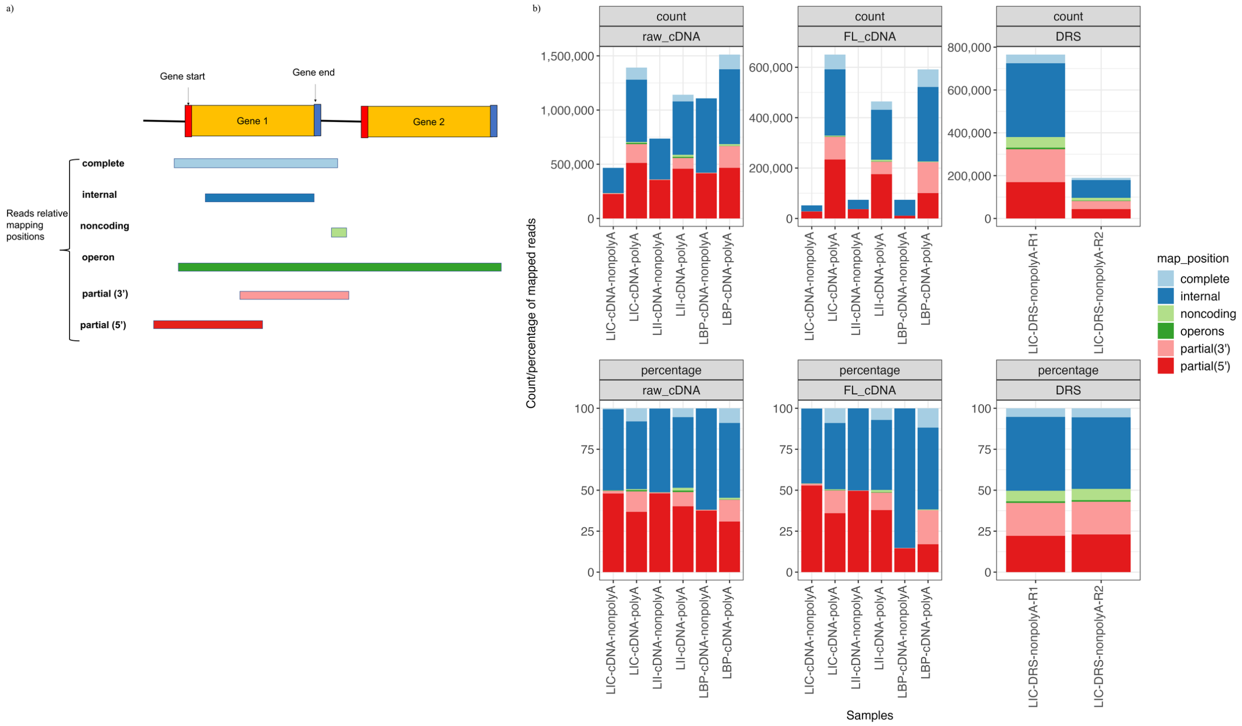


**Figure S3**. **Relative mapping position of each direct cDNA or DRS read in relation to the coordinates of the closest annotated gene it was mapped to**. (a) Diagram showing the six categories of reads mapping positions relative to their closest annotated genes in the reference genome. (b) The number and percentage of reads mapped to each mapping position category are listed in panel (a). LIC: *L. interrogans* Copenhageni; LII: *L. interrogans* Icterohaemorrhagiae; LBP: *L. biflexa* Patoc. FL: full-length; DRS: direct RNA sequencing; cDNA: direct cDNA sequencing; FL: full-length; DRS: direct RNA sequencing.

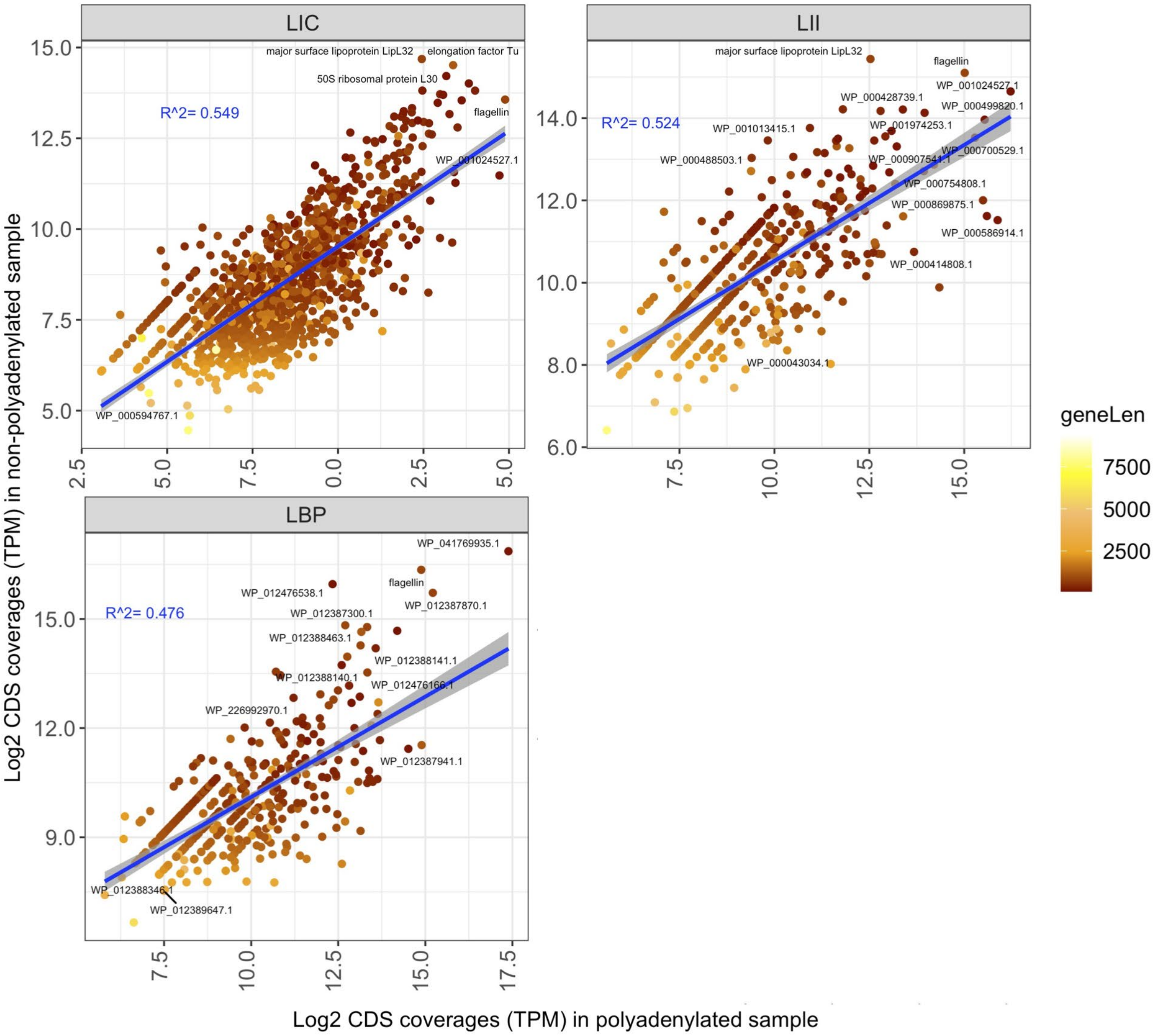


**Figure S4. Correlation of transcript coverages between non-polyadenylated and polyadenylated samples mapped by the full-length cDNA reads.** Correlation of coding region coverages (in $\log_2$ of transcript per million, TPM) between the non-polyadenylated cDNA (y-axis) and the polyadenylated cDNA sample (x-axis) mapped by the full-length cDNA reads from each strain. Each dot represents an annotated gene on the corresponding reference genome. The color of each dot is scaled by the length (in bp) of the corresponding gene. LIC: *L. interrogans* Copenhageni; LII: *L. interrogans* Icterohaemorrhagiae; LBP: *L. biflexa* Patoc.
